# Prevalence and Clinical Significance of Antiphospholipid Antibodies in Hospitalized Patients With COVID-19 Infection

**DOI:** 10.7759/cureus.27862

**Published:** 2022-08-10

**Authors:** Navkirat Kahlon, Pejma Shazadeh Safavi, Ziad Abuhelwa, Taha Sheikh, Cameron Burmeister, Sishir Doddi, Ragheb Assaly, William Barnett

**Affiliations:** 1 Hematology and Medical Oncology, The University of Toledo Medical Center, Toledo, USA; 2 Internal Medicine, The University of Toledo Medical Center, Toledo, USA; 3 Medicine, The University of Toledo College of Medicine and Life Sciences, Toledo, USA; 4 Pulmonary/Critical Care Medicine, The University of Toledo Medical Center, Toledo, USA; 5 Biostatistics and Epidemiology, The University of Toledo Medical Center, Toledo, USA

**Keywords:** c-reactive protein (crp), covid-related hypercoagulability, lymphocyte count, hospitalized, prognosis, mortality, clinical implications, prevalence, antiphospholipid antibodies, covid-19

## Abstract

Background: Coronavirus disease 2019 (COVID-19) infection is associated with an increased risk of arterial thromboembolic events (ATE) and venous thromboembolic events (VTE). Hypercoagulability associated with COVID-19 infection is multifactorial, and underlying pathogenic mechanisms potentially responsible for thrombosis include inflammation resulting in endothelial damage, platelet activation and the presence of antiphospholipid antibodies (APAs). Antiphospholipid antibody syndrome is one of the very few causes which is associated with venous and arterial thromboembolic events. COVID-19 patients have a high prevalence of APAs as well as both ATE and VTE, but their clinical significance in COVID-19 patients is not fully understood yet.

Objectives: In this study, we intend to find the prevalence of APAs in hospitalized COVID-19 patients at the time of diagnosis and determine whether their presence has any clinical significance.

Methods: This is a retrospective single-institution study involving patients hospitalized for the management of COVID-19 infection at The University of Toledo Medical Center. After obtaining approval from the biomedical institutional review board at The University of Toledo, antiphospholipid antibody (APA) testing was done on pre-stored blood samples of these patients and hospital charts were reviewed till six months from the positive COVID-19 test result. Two groups were created based on the patients' APA testing results (APA positive and APA negative) and used for statistical comparison. Any patients with positive lupus anticoagulant (LA) or abnormal titers APA antibodies were labeled as positive. Demographic data, prognostic outcomes and laboratory values were compared either using Mann-Whitney U-test for continuous variables or Fisher’s exact test for categorical variables.

Results: The prevalence of APAs in hospitalized COVID-19 patients at the time of diagnosis was 39.3% in this study. There was no difference in demographic variables between the APA-positive and APA-negative groups. The prevalence of APAs was higher in smokers, where 91% of the APA-positive patients were smokers. There was no statistically significant difference in prognostic outcomes including six-month mortality between APA-positive and APA-negative patients. The comorbidity profile was the same in the two groups. APA-positive patients were found to have lower nadir of absolute lymphocyte count and higher nadir levels of C-reactive protein during hospitalization.

Conclusions: The prevalence of APA positivity in hospitalized COVID-19 patients is higher in our study than in historical studies involving non-COVID-19 hospitalized patients, particularly in smokers. However, there is no correlation between APA positivity and prognostic outcomes including six-month mortality. At this point, it is unclear whether APAs are just bystanders or have a pathogenic role. Routine testing of APA in COVID-19 patients is not indicated. Further prospective studies to elucidate the persistence and clinical implications of APAs are needed.

## Introduction

Coronavirus disease 2019 (COVID-19) infection is associated with arterial thromboembolic events (ATE) and venous thromboembolic events (VTE); however, there is substantial variance in the incidence of such events in the literature secondary to variability in patient characteristics and study protocols. Increased prothrombin time (PT), activated partial thromboplastin time (APTT), D-dimer and fibrinogen have been used as serologic markers with sensitivity and specificity noted to be above 80th percentile and negative predictive values above 90th percentile in the detection of acute VTE [1].

Current literature supports that the hypercoagulability in COVID-19 infection is multifactorial. The proposed main factors predisposing COVID-19 patients to thrombosis include inflammation resulting in endothelial damage, platelet activation, hyperviscosity and the presence of antiphospholipid antibodies (APAs) [2]. Various underlying pathogenic mechanisms contributing to hypercoagulability have been investigated. Systemic cytokines responsible for atherosclerotic plaque rupture have been implicated in the inflammation and endovascular damage in COVID-19, which promotes a prothrombotic milieu locally [3-5]. It is also thought that angiotensin-converting enzyme 2 (expressed on myocytes and vascular endothelial cells) which acts as a receptor for COVID-19 contributes to endovascular injury [5-7]. APAs are thought to play a role in endothelial activation in COVID-19 [8].

APAs can be present in 1%-5% of the healthy population and are known to cause an autoimmune disorder called antiphospholipid syndrome (APS). APS is the most common form of acquired thrombophilia. The diagnostic criterion for APS includes the presence of both clinical criteria (recurrent venous or arterial thrombosis and/or fetal loss) and laboratory criteria (presence and persistence of APAs). Numerous APAs have been identified, but only a few are part of the diagnostic criteria for APS. The antibodies which are part of the clinical criteria for APS include lupus anticoagulant (LA), anticardiolipin antibodies (aCL) and anti-beta 2-glycoprotein I antibodies (aβ2GPI). Specifically, the immunoglobulin G (IgG) and immunoglobulin M (IgM) subclasses are included in the APS laboratory criteria [9,10]. COVID-19 patients have a high prevalence of APAs as well as both ATE and VTE, but their clinical significance in COVID-19 patients is not fully understood yet.

The presence of APAs in COVID-19 patients was brought to light in some case reports initially. In 2020, a case series of three critically ill COVID-19 patients who tested positive for APAs was reported with one patient suffering multiple bilateral cerebral infarcts and limb ischemia [11]. These cases triggered some further research regarding APAs in the context of COVID-19. APAs are known to be associated with elevated APTT. In a study involving COVID-19 patients, a subset of patients with elevated APTT was tested for the presence of APA utilizing dilute Russell’s viper venom time and lupus anticoagulant-sensitive APTT. Of those patients with elevated APTT, 91% were APA positive [10]. The clinical and laboratory data from the abovementioned studies involving COVID-19 patients seemed to suggest that APAs could be one of the contributing factors to thrombosis. As of note, antiphospholipid syndrome (primary or secondary) is one of the few causes associated with both arterial and venous thromboembolic events as seen in COVID-19 infection. All these initial findings further strengthened the interest of researchers in studying the role of APAs in COVID-19 patients. In this retrospective single-institution study, we intend to find the prevalence of APAs in hospitalized COVID-19 patients at the time of their initial presentation. Secondary objectives include determining whether the presence of APAs at initial diagnosis has any clinical significance, specifically looking at prognostic outcomes, i.e., six-month mortality, renal failure requiring dialysis, thrombotic events, need for mechanical ventilation, rehospitalizations, discharge disposition and the highest level of hospital care. Another secondary objective is to determine correlation of APAs at presentation with the known prognostic laboratory parameters for COVID-19 infection severity or laboratory parameters determining organ function.

## Materials and methods

The approval for this retrospective study conducted at the University of Toledo Medical Center was obtained from the biomedical institutional review board (IRB), IRB number: 300722-UT. Patients ≥18 years old who were hospitalized for COVID-19 infection from March 20, 2020 to June 30, 2021 and had adequate and viable pre-stored plasma and/or serum samples (per institutional policy) to test APAs were included per study protocol. There was a drop in the number of COVID-19 cases for a brief period around June 2021; so, the decision to include patients just up to this period was made. After IRB approval, a list of patients hospitalized with COVID-19 during this period was obtained from the information technology department and clinical teams’ patient lists (pulmonary and medical intensive care unit) at the University of Toledo Medical Center. Most of the patients on the list were primarily excluded due to a lack of viable blood or serum samples. Patients finally included in the study had stored blood samples available for APA testing, drawn within 48 hours (about two days) of positive COVID-19 antigen testing and hospitalization. The samples were tested for the following APAs: anticardiolipin antibodies (aCL), anti-beta 2-glycoprotein I antibodies (aβ2GPI) and lupus anticoagulant (LA). aCL and aβ2GPI testing were done on all patients. Due to a lack of adequate samples, LA testing could be done only in selected patients. Only eight patients had sufficient samples to run LA testing. The cut-off level used to define positive antibody testing was >10.9 IgG phospholipid units (GPL) for aCL immunoglobulin M (IgM), >19.9 GPL for aCL immunoglobulin G (IgG), >19.9 GPL for aβ2GPI IgM and >19.9 GPL β2GPI IgG per institutional laboratory cut-off levels. LA testing was reported as positive or negative per institutional laboratory protocol. The laboratory personnel who tested the samples were blinded to clinical data. The charts were reviewed six months after the date of the positive COVID-19 test. Once the APA panel had been run and charts were reviewed, data were coded and securely stored so that only the investigator and authorized staff could access it to ensure confidentiality. Only de-identified data were analyzed and have been reported. Two groups were created based on the patients' APA testing results, i.e., APA positive (+) and APA negative (-) and used for statistical comparison. Any patients with positive LA or abnormal titers APA antibodies were labeled as positive.

The primary objective of this study was to determine the prevalence of APAs in plasma of hospitalized COVID-19 patients. The secondary objective was to determine whether there is any correlation between the presence of APAs with adverse outcomes, i.e., six-month mortality, renal failure requiring dialysis, thrombotic events, need for mechanical ventilation, rehospitalizations, discharge disposition and the highest level of hospital care. Other secondary objectives were to determine demographic trends for APA-positive patients and the correlation between laboratory values known to be associated with poor prognosis or organ dysfunction and the presence of APAs in COVID-19 patients. Unfortunately, being a retrospective study, imaging studies such as computed tomography angiogram (CTA) or venous Doppler ultrasound were not available for most of these patients. As a result, we were unable to detect and report the presence or absence of asymptomatic thrombosis which would have been an important adverse effect of interest.

Outcomes and laboratory values were compared using non-parametric methods due to the small sample size and non-normality of the data. The Mann-Whitney U-test was used for continuous variables and Fisher’s exact test for categorical variables. All analyses were performed using R 4.0.3 (R Foundation for Statistical Computing, Vienna, Austria), and an alpha level of 0.05 was considered statistically significant.

## Results

A total of 28 patients were included in the final analysis. Seventeen of 28 patients (60.7%) were negative for APA, and 11 of 28 patients (39.3%) were positive for APA. Seven of 28 patients (25%) had positive aCL IgM with titers ranging from 11.4 to 28.1 GPL for aCL IgM-positive patients. Three of 28 patients (10.71%) had positive aCL IgG with titers ranging from 21.8 to 25.4 GPL for aCL IgG-positive patients. aβ2GPI IgM was present in two of 28 patients (7.14%) with titers ranging from 21.1 to 43.8 GPL for aβ2GPI IgM-positive patients. β2GPI IgG was not seen in any patients. LA testing could be done only in eight patients, and four of eight patients (50% of patients tested for LA, 14.2% of total number of patients) tested positive. Four of 28 patients (14.2%) had more than one positive APA test. No patient had a triple positive profile, i.e., positive testing for all three APAs (LA, aCL and aβ2GPI).

There was no difference in the selected demographic characteristics of APA-positive and APA-negative patients. The median age, gender distribution and racial/ethnic distribution were statistically similar between the two groups. The median age was 66 years (interquartile range (IQR): 53.00-68.00 years) for APA-negative patients and 60 years (IQR: 59.00-65.50 years) for APA-positive patients (p = 0.925). There was no statistically significant difference in the racial or ethnic distribution in APA-negative and APA-positive groups. Among APA-negative patients, 35.3% were White, 52.9% were Black, and 5.9% were Hispanic; among APA-positive patients, 54.5% were White, 36.4% were Black, and 0% were Hispanic (p = 0.763). The selected comorbidity profile which is commonly associated with vascular events was also similar between the two groups. In APA-negative and APA-positive groups, the past medical history of coronary artery disease was 29.4% and 18.2% (p = 0.668), atrial fibrillation was 29.4% and 18.2% (p = 0.668), stroke was 17.6% and 45.4% (p = 0.120), peripheral artery disease was 23.5% and 27.3% (p = 1.000) and cancer was 23.5% and 18.2% (p = 1.000), respectively. A statistically significant association between smoking history and APA positivity status was noted in our study. Ninety-one percent of patients who tested positive for APAs were smokers as compared to only 30% in APA-negative group. These results are summarized in Table 1.

**Table 1:** Demographics and comorbidity profile of hospitalized COVID-19 patients according to APA status APA: antiphospholipid antibodies; No.: number; IQR: interquartile range; M: male; CAD: coronary artery disease; Afib: atrial fibrillation; CVA: cerebrovascular accident; PAD: peripheral artery disease; Y: yes. ⁺Statistically significant.

Patient Demographics and Comorbidities	APA-	APA+	p-Value
No. of patients	17	11	
Age in years (median (IQR))	66.00 (53.00-68.00)	60.00 (59.00-65.50)	0.925
Sex = M (%)	9 (52.9)	9 (81.8)	0.226
Race/ethnicity (%)			0.763
White	6 (35.3)	6 (54.5)	
Black	9 (52.9)	4 (36.4)	
Hispanic	1 (5.9)	0 (0.0)	
Unknown	1 (5.9)	1 (9.1)	
Comorbidities			
CAD = Y (%)	5 (29.4)	2 (18.2)	0.668
Afib = Y (%)	5 (29.4)	2 (18.2)	0.668
CVA = Y (%)	3 (17.6)	5 (45.5)	0.120
PAD = Y (%)	4 (23.5)	3 (27.3)	1.000
Cancer = Y (%)	4 (23.5)	2 (18.2)	1.000
Smoking = Y (%)	5 (29.4)	10 (90.9)	0.002⁺

There was no statistically significant difference in prognostic outcomes of interest between APA-positive and APA-negative groups, defined as a p-value <0.05. Six-month mortality in APA-negative and APA-positive patients was similar, i.e., 35.3% and 45.5%, respectively (p = 0.701). The rates of need for intubation and hemodialysis during hospitalization for COVID-19 infection were also similar. During the hospital stay for COVID-19 infection, in APA-negative and APA-positive groups, intubation was required in 58.8% and 63.6% of patients, respectively (p = 1.000), and hemodialysis was required in 17.6% and 18.2% of patients, respectively (p = 1.000). The rehospitalization rates were also similar between the two groups, i.e., 23.5% and 36.4% in APA-negative and APA-positive patients, respectively (p = 0.707). In APA-negative and APA-positive groups, 58.8% and 90.9% of patients required ICU admission, respectively, but the difference was not statistically significant (p = 0.099). The pattern of discharge disposition was also similar between APA-negative and APA-positive groups with in-hospital mortality rates of 35.3% and 36.4%, rates of discharge to a facility of 17.6% and 36.4%, rates of discharge to a group home of 11.8% and 0.0% and rates of discharge to the home of 35.3% and 27.3%, respectively (p = 0.599). These results are summarized in Table 2.

**Table 2:** Prognostic outcomes in hospitalized patients COVID-19 patients according to APA status APA: antiphospholipid antibodies; No.: number; Y: yes; Vent: ventilator; HD/CVVHD: hemodialysis/continuous veno-venous hemodialysis; ICU: intensive care unit. *Alive at six months after diagnosis. ^#^Need for endotracheal intubation/ventilator during hospitalization for COVID-19 infection. ^Need for new dialysis secondary to renal failure during hospitalization for COVID-19 infection.

Prognostic Outcomes	APA-	APA+	p-Value
No. of patients	17	11	
Alive* = Y (%)	11 (64.7)	6 (54.5)	0.701
Vent^#^ = Y (%)	10 (58.8)	7 (63.6)	1.000
HD/CVVHD^ = Y (%)	3 (17.6)	2 (18.2)	1.000
Rehospitalization (%)			0.707
None	13 (76.5)	7 (63.6)	
Once	3 (17.6)	2 (18.2)	
Twice	1 (5.9)	2 (18.2)	
Highest level of care (%)			0.099
Intermediate	7 (41.2)	1 (9.1)	
ICU	10 (58.8)	10 (90.9)	
Disposition (%)			0.599
Death	6 (35.3)	4 (36.4)	
Facility	3 (17.6)	4 (36.4)	
Group home	2 (11.8)	0 (0.0)	
Home	6 (35.3)	3 (27.3)	

The laboratory values of interest were compared in APA-positive and APA-negative patients. There was no difference in median nadir and peak levels of hemoglobin (Hgb) during hospitalization for COVID-19 infection between the two groups. The median peak Hgb level was 12.40 grams per deciliter (g/dl) (IQR: 10.50-13.60 g/dl) in APA-negative patients in comparison to 11.30 g/dl (IQR: 10.70-13.10 g/dl) in APA-positive patients (p = 0.424). The median Hgb level nadir was 8.20 g/dl (IQR: 7.10-11.00 g/dl) in APA-negative patients in comparison to 9.50 g/dl (IQR: 7.10-10.00 g/dl) in APA-positive patients (p = 0.906). The median peak and nadir levels of median white blood cell (WBC) count were also similar. The median WBC count nadir was 5.21 x 1,000 cells per cubic millimeter of blood (/mm^3^) (IQR: 4.25-7.40 x 1,000 cells/mm^3^) vs 7.00 x 1,000 cells/mm^3^ (IQR: 4.50-9.29 x 1,000 cells/mm^3^) in APA-negative and APA-positive patients, respectively (p = 0.438). The median peak WBC count was 17.34 x 1,000 cells/mm^3^ (IQR: 13.60-23.67 x 1,000 cells/mm^3^) vs 16.46 x 1,000 cells/mm^3^ (IQR: 12.62-20.35 x 1,000 cells/mm^3^) in APA-negative and APA-positive patients, respectively. There was no difference in nadir and peak levels of platelets (plt) between the two groups either. The median peak plt level was 311.00 x 1,000 cells microliter of blood (/µl) (IQR: 217.00-368.00 x 1,000 cells/µl) in APA-negative patients in comparison to 364.00 x 1,000 cells/µl (IQR: 346.00-432.50 x 1,000 cells/µl) in APA-positive patients (p = 0.115). The median plt level nadir was 146.00 x 1,000 cells/µl (IQR: 112.00-172.00 x 1,000 cells/µl) in APA-negative patients in comparison to 182.00 x 1,000 cells/µl (IQR: 115.00-207.00 x 1,000 cells/µl) in APA-positive patients (p = 0.480). The median nadir of absolute neutrophil count (ANC) was similar between two groups, i.e., 2.80 x 1,000 cells/mm^3^ (IQR: 1.20-4.40 x 1,000 cells/mm^3^) vs 6.05 x 1,000 cells/mm^3^ (IQR: 4.10-7.47 x 1,000 cells/mm^3^) in APA-negative and APA-positive patients, respectively (p = 0.067). The median nadir of absolute lymphocyte count (ALC) was lower in APA-positive patients as compared to APA-negative patients, i.e., 0.40 x 1,000 cells/mm^3^ (IQR: 0.30-0.55 x 1,000 cells/mm^3^) vs 1.00 x 1,000 cells/mm^3^ (IQR: 0.55-1.30 x 1,000 cells/mm^3^) in APA-positive and APA-negative patients, respectively (p = 0.067).

The median peak bilirubin level was similar between the two groups, i.e., 0.65 milligrams per deciliter (mg/dl) (IQR: 0.48-0.85 mg/dl) vs 0.50 mg/dl (IQR: 0.40-0.75 mg/dl) in APA-negative and APA-positive patients, respectively (p = 0.281). The median albumin level nadir was also similar, i.e., 2.75 grams per deciliter (g/dl) (IQR: 2.08-3.12 g/dl) vs 2.70 g/dl (IQR: 2.35-3.25 g/dl) in APA-negative and APA-positive patients, respectively (p = 0.843). The nadir of C-reactive protein (CRP) levels was in a higher range in APA-positive patients than in APA-negative patients, i.e., 73.35 mg/dl (IQR: 46.85-87.22 mg/dl) vs 22.45 mg/dl (IQR: 13.15-73.30 mg/dl) in APA-positive and APA-negative patients, respectively (p = 0.015). However, the median peak CRP levels were not different between the two groups, i.e., 190.00 mg/dl (IQR: 119.00-240.50 mg/dl) vs 131.00 mg/dl (IQR: 95.18-281.75 mg/dl) in APA-negative and APA-positive patients, respectively (p = 0.772). Other inflammatory marker levels such as ferritin, erythrocyte segmentation rate (ESR) and D-dimer were similar between the two groups. The median peak ESR was 47.00 mm/hr (IQR: 27.50-73.50 mm/hr) vs 85.00 mm/hr (IQR: 50.00-102.00 mm/hr) in APA-negative and APA-positive patients, respectively. The median peak D-dimer level was 5.03 microgram/milliliter (μg/ml) (IQR: 1.42-8.22 μg/ml) in APA-negative patients in comparison to 3.57 μg/ml (IQR: 1.77-4.12 μg/ml) in APA-positive patients (p = 0.734). The median peak ferritin level was 1173.00 microgram/liter (µg/l) (IQR: 372.00-2688.50 µg/l) in APA-negative patients in comparison to 793.00 μg/l (IQR: 173.50-783.00 μg/l) in APA-positive patients (p = 0.736). The median ferritin level nadir was 619.00 µg/l (IQR: 173.50-783.00 µg/l) in APA-negative patients in comparison to 793.00 μg/l (IQR: 347.50-1419.00 μg/l) in APA-positive patients (p = 0.223). The creatinine peak and lactate dehydrogenase (LDH) peak levels were also similar in the two groups. The median peak lactate dehydrogenase (LDH) level was 444.00 international units per liter (IU/l) (IQR: 292.00-700.00 IU/l) in APA-negative patients in comparison to 436.00 IU/l (IQR: 274.00-621.00 IU/l) in APA-positive patients (p = 0.881). The median creatinine level peak was 1.60 mg/dl (IQR: 1.23-3.68 mg/dl) in APA-negative patients in comparison to 1.58 mg/dl (IQR: 1.26-5.66 mg/dl) in APA-positive patients (p = 0.621). These results are summarized in Table 3.

**Table 3:** Laboratory data of the hospitalized COVID-19 patients according to APA status APA: antiphospholipid antibodies; No.: number; Y: yes; Hgb: hemoglobin in grams per deciliter (g/dl); IQR: interquartile range; WBC: white blood cell count in cells x 1,000 per cubic millimeter of blood (/mm^3^); PLT: platelets count x 1,000 per microliter of blood (/µl); ANC: absolute neutrophil count x 1,000 cells per cubic millimeter of blood (/mm^3^); ALC: absolute lymphocyte count x 1,000 cells per cubic millimeter of blood (/mm^3^); Bili: bilirubin in milligrams per deciliter (mg/dl) during hospitalization for COVID-19 infection; Alb: albumin in grams per deciliter (g/dl) during hospitalization for COVID-19 infection; ESR: erythrocyte sedimentation rate in millimeters/hour (mm/hr) during hospitalization for COVID-19 infection; CRP: C-reactive protein in mg/dl during hospitalization for COVID-19 infection; LDH: lactate dehydrogenase in international units per liter (IU/l) during hospitalization for COVID-19 infection; Cr: creatinine in mg/dl during hospitalization for COVID-19 infection. *D-dimer levels in microgram/milliliter (μg/ml) and ferritin levels in microgram/liter (µg/l). ⁺ Statistically significant.

Laboratory Values	APA-	APA+	p-Value
No. of patients	17	11	
Peak Hgb (median (IQR))	12.40 (10.50-13.60)	11.30 (10.70-13.10)	0.424
Hgb nadir (median (IQR))	8.20 (7.10-11.00)	9.50 (7.10-10.00)	0.906
WBC nadir (median (IQR))	5.21 (4.25-7.40)	7.00 (4.50-9.29)	0.438
Peak WBC (median (IQR))	17.34 (13.60-23.67)	16.46 (12.62-20.35)	0.832
Peak PLT (median (IQR))	311.00 (217.00-368.00)	364.00 (346.00-432.50)	0.115
PLT nadir (median (IQR))	146.00 (112.00-172.00)	182.00 (115.00-207.00)	0.480
ANC nadir (median (IQR))	2.80 (1.20-4.40)	6.05 (4.10-7.47)	0.067
ALC nadir (median (IQR))	1.00 (0.55-1.30)	0.40 (0.30-0.55)	0.026⁺
Peak bili (median (IQR))	0.65 (0.48-0.85)	0.50 (0.40-0.75)	0.281
Alb nadir (median (IQR))	2.75 (2.30-3.12)	2.70 (2.35-3.25)	0.843
Peak ESR (median (IQR))	47.00 (27.50-73.50)	85.00 (50.00-102.00)	0.186
Peak D-dimer* (median (IQR))	5.03 (1.42-8.22)	3.57 (1.77-4.12)	0.734
Peak ferritin* (median (IQR))	1173.00 (372.00-2688.50)	793.00 (473.50-2402.00)	0.736
Ferritin nadir* (median (IQR))	619.00 (173.50-783.00)	793.00 (347.50-1419.00)	0.223
CRP nadir (median (IQR))	22.45 (13.15-73.30)	73.35 (46.85-87.22)	0.015⁺
Peak CRP (median (IQR))	190.00 (119.75-240.50)	131.00 (95.18-281.75)	0.772
Peak LDH (median (IQR))	444.00 (292.00-700.00)	436.00 (274.00-621.00)	0.881
Peak Cr (median (IQR))	1.60 (1.23-3.68)	1.58 (1.26-5.66)	0.621

## Discussion

APAs have been found to be associated with COVID-19 patients; however, data regarding their persistence and clinical implications are limited. LA- and aCL-positive titers have also been associated with other infections such as hepatitis C, cytomegalovirus (CMV), Epstein-Barr virus (EBV), adenovirus, parvovirus and human immunodeficiency virus (HIV) [12]. In our study, the prevalence of APAs was found to be 39.3% in hospitalized COVID-19-positive patients when tested on samples collected within 48 hours of hospital admission for the management of COVID-19 infection. A previous study involving over a thousand patients showed an APA positivity prevalence of 7.1% in hospitalized patients [13]. Hospitalized COVID-19 patients have a higher prevalence of APAs as compared to historic studies involving hospitalized patients without COVID-19 infection.

Looking at APA subgroups, our study found that 25% of the hospitalized COVID-19 patients had positive aCL IgM, 10.71% had positive aCL IgG, 7.14 % had positive aβ2GPI IgM and 50% of the tested patients had positive LA. β2GPI IgG was not seen in any patients. About 14.2% of patients had more than one positive APA test. No patient had a triple positive (positive testing for all three APAs) profile. A systematic review involving COVID-19 patients found the prevalence of LA ranging from 35% to 92% in intensive care unit (ICU) patients; aCL IgG and IgM up to 52% and up to 40% of patients, respectively; and aβ2GPI IgG and IgM up to 39% and up to 34% of patients, respectively. One percent and 12% of patients were found to have a triple positive APA profile [14]. These numbers corresponding to APA subgroups in the systematic review are higher than in our study. However, the systematic review included only ICU patients, and our study included all hospitalized patients. This difference in results between these two studies could potentially indicate a higher prevalence of APAs in ICU patients than in all hospitalized patients.

There was no difference in demographic profile or comorbidity profile in APA-positive and APA-negative patients in our study. A statistically significant association between smoking history with APA positivity status was noted in our study. Ninety-one percent of patients who tested positive for APAs were smokers as compared to only 30% of patients in the APA-negative group. An association between smoking and APA positivity has been demonstrated in other studies involving non-COVID-19 patients too, and a higher risk of vascular events in these patients has been demonstrated [15,16]. If we extrapolate data from non-COVID-19 patients, it is possible that APA positivity may be associated with a higher risk of vascular events in hospitalized COVID-19 patients with a smoking history as well, but this hypothesis cannot be tested in our study given the small number of patients and the retrospective nature of the study. The smokers with COVID-19 infection must be studied in prospective studies to determine the prognostic and predictive role of APAs in this patient population specifically.

The thromboembolic events including VTE and ATE have been reported in COVID-19 patients. These thromboembolic events in COVID-19 are associated with high mortality [17]. Although high titers of APAs in COVID-19 patients have been seen, the correlation between thrombotic events or mortality and APA positivity in COVID-19 remains under investigation. So far, no association between APA positivity and in-hospital mortality has been found [18]. There are hardly any studies that looked at the presence of APA positivity and long-term mortality in COVID-19 patients which was evaluated in our study. We found that there was no difference in long-term outcomes, specifically six-month mortality between the APA-positive or APA-negative patients. Also, there was no difference in prognostic outcomes determining disease severity in this study, i.e., in-hospital mortality, rehospitalizations, need for mechanical ventilation, hemodialysis or discharge disposition to a facility between these two groups. In contrast, a previous longitudinal study done by Trahtemberg et al. involving APA-positive patients with respiratory failure admitted to intensive care involving COVID-19-positive and COVID-19-negative patients found that positive APA serology had an association with more severe disease regardless of COVID-19 status [19]. This difference in results might be due to the difference in patients included in the two studies. Our study included all hospitalized patients, and the study by Trahtemberg et al. included a sicker ICU patient population [19]. Trahtemberg et al. did not find any association between APA positivity and increased thrombotic risk [19]. Unfortunately, the retrospective data from our study did not allow us to assess VTE risk in these patients as most patients did not have imaging studies done to assess VTE.

We compared the selected laboratory values between APA-positive and APA-negative patient groups. In our study, there was no correlation between APA serum positivity and most of the hematologic parameters, i.e., peak and nadir hemoglobin level, platelet count, total leukocyte count and absolute neutrophil count. The only exception was the lower nadir of absolute lymphocyte count (ALC) in APA-positive patients. As of note, the low lymphocyte count is a known poor prognostic marker for COVID-19 infection and has an association with increased COVID-19 mortality [20]. However, our study demonstrates lower ALC in APA-positive group; there was no association with increased mortality in this group. A single-arm meta-analysis from this study comparing non-severe and severe COVID-19 cases showed severe cases that had lower platelet counts (165.12 in severe cases, 190.09 in non-severe cases), D-dimer (0.49 in severe cases, 0.27 in non-severe cases) and fibrinogen (4.34 in severe cases, 3.19 in non-severe cases) [21]. There was no correlation between APA positivity and these laboratory parameters associated with severe COVID-19 infection, i.e., D-dimer levels, low platelet levels and high fibrinogen levels. The inflammatory markers such as ferritin and ESR were also similar. The nadir of CRP levels was higher in APA-positive patients suggesting more inflammation in this subgroup. The laboratory values determining organ dysfunction and hence the disease severity such as creatinine level, bilirubin, LDH and albumin were similar in the two groups.

We acknowledge the limitations of our study. One limitation of our study is the small sample size. It was primarily limited by a number of patients with available viable serum samples for testing APA levels. We do not have serial APA levels as this is not a prospective study. Being a retrospective study, we did not have imaging studies such as CTA or venous Doppler ultrasound available for most of these patients to detect and report the presence or absence of asymptomatic thrombosis which would have been an important adverse effect of interest. Also, confounding variables cannot be adjusted due to the study’s retrospective nature. Post hoc power analysis was computed on the significant variables found in the study. While the association between smoking status and APA positivity was well-powered (95.8%), it is likely that the results from APA positivity and CRP nadir as well as ALC nadir association are underpowered in our study (35.5% and 64.8%, respectively).

## Conclusions

The prevalence of APA positivity in hospitalized COVID-19 patients is higher in our study than in historical studies involving non-COVID-19 hospitalized patients, particularly in smokers. However, there is no correlation between APA positivity and disease severity. Also, six-month mortality in APA-positive patients is the same as in APA-negative patients. At present, it remains unclear whether these antibodies are just bystanders or play a pathogenic role in COVID-19-related hypercoagulability. We do not recommend routine testing of APAs in COVID-19 patients. However, further prospective studies to elucidate the persistence and clinical implications of APAs are needed, particularly in smokers.
